# Lysozyme M deficiency leads to an increased susceptibility to *Streptococcus pneumoniae*-induced otitis media

**DOI:** 10.1186/1471-2334-8-134

**Published:** 2008-10-08

**Authors:** Jun Shimada, Sung K Moon, Haa-Yung Lee, Tamotsu Takeshita, Huiqi Pan, Jeong-Im Woo, Robert Gellibolian, Noboru Yamanaka, David J Lim

**Affiliations:** 1 The Gonda Department of Cell and Molecular Biology, House Ear Institute, Los Angeles, CA, USA; 2 Department of Otolaryngology - Head and Neck Surgery, School of Medicine, Wakayama Medical University, Wakayama, Japan; 3 Department of Otorhinolaryngology, Hamamatsu University School of Medicine, Hamamatsu, Japan; 4 Department of Otolaryngology, University of Southern California, Los Angeles, CA, USA; 5 Department of Cell and Neurobiology, Keck School of Medicine, University of Southern California, Los Angeles, CA, USA

## Abstract

**Background:**

Lysozyme is an antimicrobial innate immune molecule degrading peptidoglycan of the bacterial cell wall. Lysozyme shows the ubiquitous expression in wide varieties of species and tissues including the tubotympanum of mammals. We aim to investigate the effects of lysozyme depletion on pneumococcal clearance from the middle ear cavity.

**Methods:**

Immunohistochemistry was performed to localize lysozyme in the Eustachian tube. Lysozyme expression was compared between the wild type and the lysozyme M^-/- ^mice using real time quantitative RT-PCR and western blotting. Muramidase activity and bactericidal activity of lysozyme was measured using a lysoplate radial diffusion assay and a liquid broth assay, respectively. To determine if depletion of lysozyme M increases a susceptibility to pneumococal otitis media, 50 CFU of *S. pneumoniae *6B were transtympanically inoculated to the middle ear and viable bacteria were counted at day 3 and 7 with clinical grading of middle ear inflammation.

**Results:**

Immunolabeling revealed that localization of lysozyme M and lysozyme P is specific to some/particular cell types of the Eustachian tube. Lysozyme P of lysozyme M^-/- ^mice was mainly expressed in the submucosal gland but not in the tubal epithelium. Although lysozyme M^-/- ^mice showed compensatory up-regulation of lysozyme P, lysozyme M depletion resulted in a decrease in both muramidase and antimicrobial activities. Deficiency in lysozyme M led to an increased susceptibility to middle ear infection with *S. pneumoniae *6B and resulted in severe middle ear inflammation, compared to wild type mice.

**Conclusion:**

The results suggest that lysozyme M plays an important role in protecting the middle ear from invading pathogens, particularly in the early phase. We suggest a possibility of the exogenous lysozyme as an adjuvant therapeutic agent for otitis media, but further studies are necessary.

## Background

Otitis media (OM), or middle ear infection is one of the most common pediatric infectious diseases, second only to the common cold and the most common cause of hearing impairment in children [1,2]. It is not a life-threatening disease, but its socioeconomic impact is significant [3]. The rapid worldwide increase of antibiotic resistance among OM pathogens such as *S. pneumoniae *and nontyepable *H. influenzae *has given rise to an urgent need to develop new non-antibiotic approaches to prevent and manage this disease [4].

OM occurs when OM pathogens of the nasopharynx enter the middle ear via the Eustachian tube (E-tube) [5]. E-tube is covered by mucociliary epithelium as a part of the upper respiratory tract, connecting the middle ear cavity to the nasopharynx. It plays an important role in the ventilation and protection of the middle ear cavity [6]. Under certain circumstances, when these microorganisms colonize nasopharyngeal mucosal surface as commensal bacteria, they may gain access to the middle ear cavity through the E-tube, resulting in OM [7,8]. Defense of the E-tube and middle ear (tubotympanum) against invading pathogens is provided by numerous factors including the mucociliary system and the antimicrobial molecules of the innate and adaptive immune systems [9].

Lysozyme, extensively studied as one of the antimicrobial innate immune molecules (AIIMs), is ubiquitously synthesized and secreted by glandular serous cells, surface epithelial cells, and macrophages in the human airway [10-12]. In the tubotympanum, lysozyme is found in specialized epithelial cells, including those of the serous glands of the E-tube and middle ear mucosa [9,13]. Mice have two lysozyme genes [14]: 1) lysozyme M predominantly expressed in macrophage, bone marrow and lung tissue, and 2) lysozyme P mainly expressed at high levels in the small intestine (Paneth cells) and at much lower or undetectable levels in other cells/tissues. [15]. Only lysozyme M gene targeted animals are available as a model of lung and skin infection [16,17]. We aimed to assess the muramidase activity and the antimicrobial property of lysozyme in the E-tube of lysozyme M^-/- ^mice in order to evaluate the role of lysozyme in OM pathogenesis. In this study, we show for the first time that depletion of lysozyme results in delayed clearance of *S. pneumoniae *from the middle ear cavity.

## Methods

### Bacterial culture

*Streptococcus pneumoniae *serotype 6B was purchased from ATCC (Manassas, VA). The bacteria were plated on chocolate agar plate and incubated overnight at 37°C in 5% CO_2_. A single colony was inoculated in 10 ml of Todd-Hewitt broth (THB, Becton Dickinson, Cockeysville, MD) and incubated overnight. One ml of this overnight culture was then transferred to 9 ml of fresh medium and incubated for a further 3 h. The bacteria were washed twice with 10 mM sodium phosphate and the optical density (O.D.) at 620 nm was determined after resuspension in sterile saline.

### Animals and the middle ear infection model

Lysozyme M^-/- ^mice were generously provided by Dr. Tomas Ganz (University of California, Los Angeles, CA). Lysozyme M-deficient mice (in which green fluorescent protein was knocked into the lysozyme M locus) were generated as previously described [18]. Since the resulting transgenic mice contained contributions from both C57BL/6 and 129Sv strains the non-transgenic parental strain C57BL/6 was chosen as a control mouse. All mice were housed under SPF conditions and all aspects of animal handling were performed according to the approved IACUC guidelines.

In total 56 eight-week old mice of C57BL/6 and lysozyme M^-/- ^mice were used in this study. Four mice were used for immunolabeling, 12 mice for protein and RNA extraction, and 40 mice for the infection study. Animals were anesthetized intraperitoneally, with a cocktail of 20 mg/ml of ketamine-HCl and 2.5 mg/ml of xylazine. Similar to a previously described study [19], we determined the optimum concentration of *S. pneumoniae *that causes substantial middle ear inflammation without mortality in wild type mice. The middle ear was transtympanically inoculated with 50 CFU of *S. pneumoniae *6B in 5 μl of sterile saline. The tympanic membrane was observed on the 1^st^, 3^rd^, 5^th ^and 7^th ^day after inoculation using an otoscopic digital imaging system, MedRx VetScope System^® ^(MedRx Inc., Largo, FL). Tympanic membrane inflammation was graded on a scale of 0 to 2 as follows; Grade 0: normal, Grade 1: mild inflammation with air-fluid level and Grade 2: severe inflammation with massive middle ear effusion. Middle ear lavage was collected on day 3 and day 7 by washing with 5 μl of sterile saline for four times and was subsequently plated on chocolate agar plates. Colonies were counted after overnight incubation. To confirm the recovered bacteria, PCR was performed using the specific primers to *S. pneumoniae *(5'-TGTTGCCAGCTTGTTCTTAGGAGGA-3', 5'-TTTTGACGTCAACTCAGCTTCCGAT-3') [20].

### Immunolabeling

Immunolabeling was performed to assess the expression profile of lysozyme in the mouse E-tube. 8-week-old lysozyme M^-/- ^and age-matched wild type mice were sacrificed, and the temporal bones were removed and fixed in 4% paraformaldehyde in PBS. After decalcification in 125 mM EDTA in PBS for 2 weeks, the temporal bones were dehydrated and embedded in paraffin. Six μm-thick sections were prepared and mounted on resin-coated slides. For antigen retrieval, pre-heated citrate buffer (0.01 M, pH 6.0) was applied for 15 min at 37°C. 3% hydrogen peroxide solution was applied for 10 min to block endogenous peroxidase activity. After blocking with 10% non-immune goat serum for 30 min, the sections were incubated with 1:1000 rabbit polyclonal anti-human lysozyme antibody (EC 3.2.1.17) (DAKO, Carpinteria, CA) overnight at 4°C. After washing three times with PBS, the biotinylated secondary antibody was applied for 30 min at room temperature. After peroxidase was attached by the avidin/biotin complex method, signals were detected by 3-amino-9-ethylcarbazole (AEC) (Zymed Laboratories Inc., San Francisco, CA). For a negative control, the non-immune rabbit serum was used. Sections were viewed and photographed using a Zeiss Axiovert^® ^135 TV microscope equipped with AxioVision^® ^systems (Zeiss, Germany). To evaluate the phenotypic expression of lysozyme at different parts of the E-tube, lysozyme-positive cells of the tubal epithelium were counted and normalized to the total number of non-ciliated tubal epithelial cells since ciliated cells are lysozyme negative. The lysozyme expression pattern was shown by the ratio of the lysozyme-positive tubal epithelial cells to the total non-ciliated tubal epithelial cells.

### Real time quantitative PCR

Lung tissue and E-tube tissue were obtained from the wild type and the lysozyme M^-/- ^mice. Total RNA was isolated using TRIzol^® ^reagent (Invitrogen, Carlsbad, CA) according to the manufacturer's protocol. cDNA was generated from 1 μg of total RNA using a Superscript II-kit (Gibco BRL, Rockville, MD). Real time quantitative PCR was performed to assess lysozyme M and lysozyme P mRNA expression using 7500 Real Time PCR System (Applied Biosystems, Atlanta, GA) with SYBR^® ^Green PCR Master Mix (Applied Biosystems) as described previously [21]. The primers used in this study are as follows: lysozyme M (GenBank Accession No. AH001999): 5'-CTGGCTACTATGGAGTCAGC-3' and 5'-TTGATCCCACAGGCATTCAC-3', lysozyme P (GenBank Accession no. BC061129): 5'-CAGGCCAAGGTCTACAATCG-3' and 5'-TTGATCCCACAGGCATTCTT-3', and 18S rRNA (GenBank accession no. V012705): 5'-GTGGAGCGATTTGTCTGGTT-3' and 5'-CGCTGAGCCAGTCAG TGTAG-3'. Cycle threshold (CT) values of lysozyme M and lysozyme P were normalized to the mouse 18S rRNA internal control, and relative quantity of mRNA was determined by the 2(-DDCT) method [22]. Results were expressed as a fold-expression of mRNA quantity, taking the value of the lysozyme P mRNA in the lung as 1.

### Lysoplate radial diffusion assay for muramidase activity

The mouse E-tube was dissected after euthanization with CO_2 _inhalation. The tissue was weighed and homogenized using a Pellet Pestle^® ^Motor (Kontes Co., Vineland, NJ) with 2 volumes (v/w) of 5% acetic acid. The extract was centrifuged at 10,000 × g for 45 min and the supernatant was collected and stored at -80°C. Total protein concentration was measured using the Bio-Rad protein assay kit (Bio-Rad, Hercules, CA). 0.5 mg/ml lyophilized *Micrococcus lysodeiktikus *(Sigma, St. Louis, MO) was suspended in 66 mM sodium phosphate buffer (Na_2_HPO_4_/NaH_2_PO_4_, pH 7.4) and poured into a square Petri dish with 1% agarose in 66 mM sodium phosphate as described previously [23]. 4 mm-sized wells were made using a cork borer (WR Scientific, West Chester, PA), and filled with 6 μl of either tissue homogenates (0.17 mg/ml) or serial dilutions of chicken egg white lysozyme (Sigma) as standards. After overnight incubation at room temperature, the muramidase activity was determined by measuring the diameter of the clear zone relative to the standards.

### Gel-overlay assay

The gel-overlay assay was performed to demonstrate the activity of lysozyme fraction in the tissue homogenate as previously described [24]. Briefly, the E-tube homogenate was extracted with 5% acetic acid and vacuum-dried. After resuspension in 3 × loading dye, the E-tube homogenate (15 μg/lane) was separated on a 12.5% Acid-Urea polyacrylamide gel electrophoresis (AU-PAGE) for 1.5 h at 100 V. The gel was washed for 20 min in 10 mM sodium phosphate buffer pH 7.4 and placed on a pre-made underlay gel (0.1 × THB, 10 mM sodium phosphate, 0.8% low electroendosmosis (EEO)-type agarose) containing either 10^6 ^CFU/10 ml of *S. pneumoniae *(for antimicrobial activity) or lyophilized *M. lysodeiktikus *(for muramidase activity). After incubation of both over-lay and under-lay gels at 37°C for 3 h, the AU-PAGE (over-lay) gel was removed, and the underlay gel was further incubated overnight at 37°C in a 5% CO_2_. After staining with Coomassie Blue, the clear zones were visualized, showing antimicrobial activity and muramidase activity of lysozyme fraction in the tissue homogenate.

### Liquid broth assay

Antimicrobial activity of the E-tube homogenate was determined as described previously [25]. Briefly, one tenth of an overnight culture broth was transferred to fresh medium and allowed to grow to exponential phase. After washing with phosphate-buffered saline, bacteria were suspended in a standard assay buffer (1% THB medium -10 mM sodium phosphate, pH 7.4) and O.D was measured at 620 nm. 2.5 × 10^4 ^CFU/mL of *S. pneumoniae *was then exposed to the E-tube homogenate (100 μg/mL) or lysozyme from human milk (Sigma) or chicken egg white for 2 h at 37°C. Viability of *S. pneumoniae *was measured by plating several dilutions to the chocolate agar and counting the number of colonies after overnight incubation at 37°C.

### Western Blot Analysis

The E-tube homogenate (15 μg/lane) was separated on a 12.5% Acid-Urea polyacrylamide gel electrophoresis (AU-PAGE) for 1.5 h at 100 V. Then the gels were blotted to PVDF membranes (Invitrogen, Carlsbad, CA) for 40 min in 0.7% acetic acid and 10% methanol at 0.18 amperes. After fixation with 10% formalin in TBS (Tris-buffered saline), the membranes were blocked in 5% non-fat dry milk in TBS for 1 h at room temperature. The membranes were incubated with a 1:5,000 dilution of rabbit polyclonal anti-human lysozyme antibody (EC 3.2.1.17) (DAKO) overnight at 4°C. After washing with 1× TBST (Tris-buffered saline with 0.05% Tween 20), the membranes were incubated with a 1:3,000 dilution of HRP-conjugated anti-rabbit IgG (Cell Signaling Technology, Danvers, MA). After washing, the membranes were incubated in SuperSignal substrate (Pierce Biotechnologies) for 1 min at room temperature. The chemiluminescence signal was detected by exposure to X-ray film and quantitated using the Quantity One^® ^software (Bio-Rad Laboratories, Hercules, CA).

### Statistical Analysis

All experiments were carried out in triplicate. Results were expressed as mean ± standard deviation. Statistical analysis was performed using Student's t-test, with significance considered to be p < 0.05. Fisher's exact test was particularly performed for analyzing bacterial clearance and host inflammation data between wild type and lysozyme M^-/- ^mice, with significance considered to be p < 0.05.

## Results

### Localization of lysozyme M and lysozyme P in the E-tube

**Figure 1:**
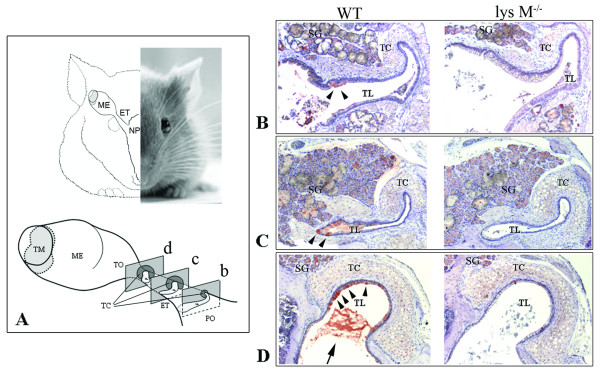
**Immunolabeling showing the phenotypic expression of lysozyme in the E-tube**. The schematic illustration showing right tympanic bulla and E-tube of a mouse (A). ME: middle ear, ET: Eustachian tube, NP: nasopharynx, TM: tympanic membrane, PO: pharyngeal orifice, TO: tympanic orifice, C: tubal cartilage, TL: E-tube lumen. Cross-sections at level b, c and d of both wild type and lysozyme M^-/- ^mice are shown in B, C and D. Lysozyme is labeled in both the submucosal gland (SG) and the E-tube epithelium (arrow heads) of wild type mice while it is labeled predominantly in the submucosal gland (SG) of lysozyme M^-/- ^mice. This indicates that lysozyme P of lysozyme M^-/- ^mice is predominantly expressed in the submucosal gland, not in the tubal epithelium, considering that the polyclonal antibody recognizes both lysozyme M and lysozyme P. The secretion in the tubal lumen (arrow) is also immunolabeled with lysozyme in wild type mice, but not in lysozyme M^-/- ^mice. All sections were immunolabeled with anti-human lysozyme antibody (1:1,000) and counterstained with hematoxylin. For each pair of images the left-hand panel displays sections from wild type mice and the right-hand displays sections from lysozyme M^-/-^mice. TC: tubal cartilage, TL: E-tube lumen. SG: submucosal gland. Original magnification: × 100.

We characterized the pattern of lysozyme expression in the E-tube of wild type and lysozyme M^-/- ^mice. Immunolabeling was performed using the polyclonal anti-human lysozyme antibody, recognizing both lysozyme M and lysozyme P. Lysozyme was widely labeled in the tubal epithelial cells and the submucosal glandular cells of the wild type mice. There were distinct differences in the distribution of the lysozyme-positive cells along the length of the E-tube. In the pharyngeal and the middle portions of the wild type mouse E-tube, lysozyme-positive cells were localized mainly in the inferior recessed portion of the luminal surface (Figure 1B, C). In the tympanic orifice, most epithelial cells showed a positive labeling with lysozyme in wild type, but virtually none in lysozyme M^-/- ^mice (Figure 1D). The secretion was noted in the tubal lumen of both mice with lysozyme-positive labeling only in the wild type mice, but not in the lysozyme M^-/- ^mice. Interestingly, the E-tube of lysozyme M^-/- ^mice showed very few lysozyme-positive cells in the tubal epithelium compared to the submucosal glands. This indicates that lysozyme P isoform in lysozyme M^-/- ^mice is predominantly expressed in the submucosal glands, and not in the tubal epithelium.

To determine the distribution of lysozyme-positive cells in the tubal epithelium, we counted total tubal epithelial cells and lysozyme-positive cells from three regions – pharyngeal orifice, middle portion and tympanic orifice – of the E-tube (Table 1). There was no significant difference in the distribution of the lysozyme-positive tubal epithelial cells along the length of the E-tube in the wild type mice. The lysozyme-positive tubal epithelial cells were significantly decreased in lysozyme M^-/- ^mice at all three portions of the E-tube, compared to the wild type mice.

**Table 1:** Distribution of lysozyme-positive cells in the E-tube epithelium.

	Distribution of lysozyme-positive cells (%)*		
	
Mouse	Pharyngeal orifice	Middle portion	Tympanic orifice
Wild type	37.8 ± 22.0	39.0 ± 5.8	45.4 ± 2.5
Lysozyme M^-/-^	0	2.6 ± 2.3	0.5 ± 0.9
	(*p *< 0.05)	(*p *< 0.01)	(*p *< 0.01)

*: The number of lysozyme-positive cells was divided by total number of non-ciliated epithelial cells. Values are given as the mean ± standard deviation (n = 3).

### Lysozyme P in the E-tube of lysozyme M^-/- ^mice

**Figure 2:**
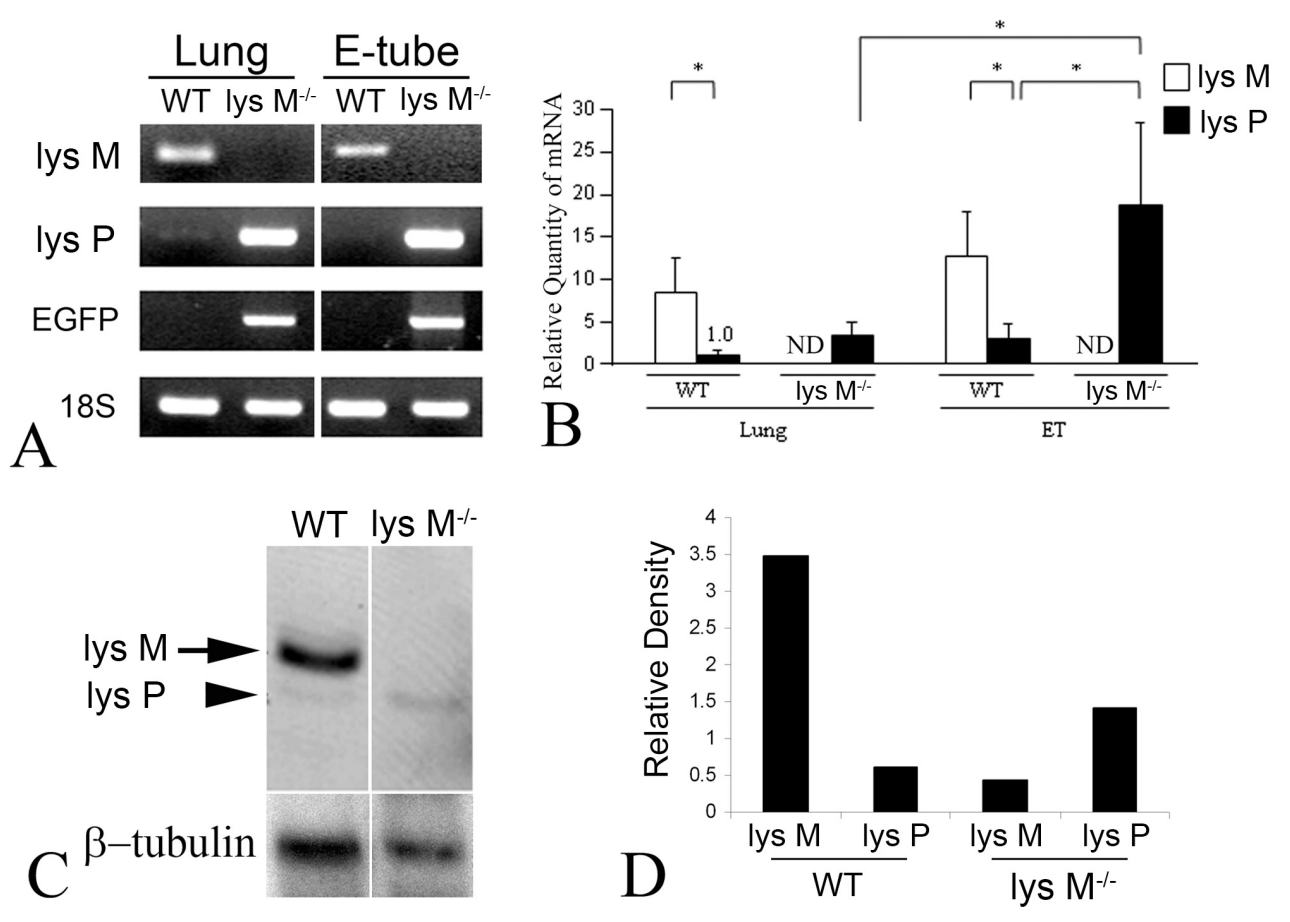
**Compensatory up-regulation of lysozyme P in lysozyme M^-/- ^mice**. Conventional RT-PCR shows compensatory up-regulation of lysozyme P when substituting the lysozyme M gene with EGFP in lysozyme M^-/- ^mice (A). Real time quantitative RT-PCR analysis shows that lysozyme P is remarkably up-regulated in the E-tube of lysozyme M^-/- ^mice, compared to the lung (B). ND: not detected. Results were expressed as a fold-expression of mRNA quantity, taking the value of lysozyme P mRNA of the lung as 1. Values are given as mean ± standard deviation. n = 3. *: p < 0.05. The western blot (C) and its densitometry (D) show the expression of lysozyme protein in the E-tube homogenate. As expected, lysozyme M protein (arrow) is depleted and lysozyme P protein (arrow head) is up-regulated in the E-tube of lysozyme M^-/- ^mice, compared to wild type mice. The soluble protein fraction of the E-tube homogenate was separated using 12.5% AU-PAGE electrophoresis, subsequently transferred onto PVDF membranes and labeled with a polyclonal anti-lysozyme antibody (1:5000). Signal was detected by exposure to X-ray film and quantified by the Quantity One^® ^software.

It is reported that lysozyme P is normally inactive in macrophages, but knockout of the lysozyme M gene induces lysozyme P expression [16]. To determine the compensatory up-regulation of lysozyme P in the E-tube and the lung of lysozyme M^-/- ^mice, conventional RT-PCR was performed. The results showed compensatory up-regulation of lysozyme P when substituting lysozyme M gene with EGFP in lysozyme M^-/- ^mice (Figure 2A). We then performed real time quantitative PCR to measure the relative quantity of lysozyme M and lysozyme P mRNA. Taking the value of the lysozyme P mRNA of the lung as 1, lysozyme P mRNA was remarkably up-regulated in the E-tube of lysozyme M^-/- ^mice, compared to the lung (Figure 2B). In the lysozyme M^-/- ^mice, lysozyme P mRNA of the E-tube was unexpectedly up-regulated more than 6 folded, compared to that of the lung.

We sought to explore the lysozyme protein expression in the E-tube. The soluble protein fraction of the E-tube homogenate was separated using 12.5% AU-PAGE electrophoresis and subsequently transferred onto PVDF membranes and labeled with a polyclonal anti-human lysozyme antibody (1:5000), recognizing both lysozyme M and lysozyme P proteins. As shown in Figure 2C and 2D, the western blot and its densitometry demonstrated that lysozyme M protein is depleted and lysozyme P protein is up-regulated in the E-tube of lysozyme M^-/- ^mice, compared to wild type mice.

### Muramidase activity and antimicrobial activity of lysozyme M^-/- ^mice

**Figure 3:**
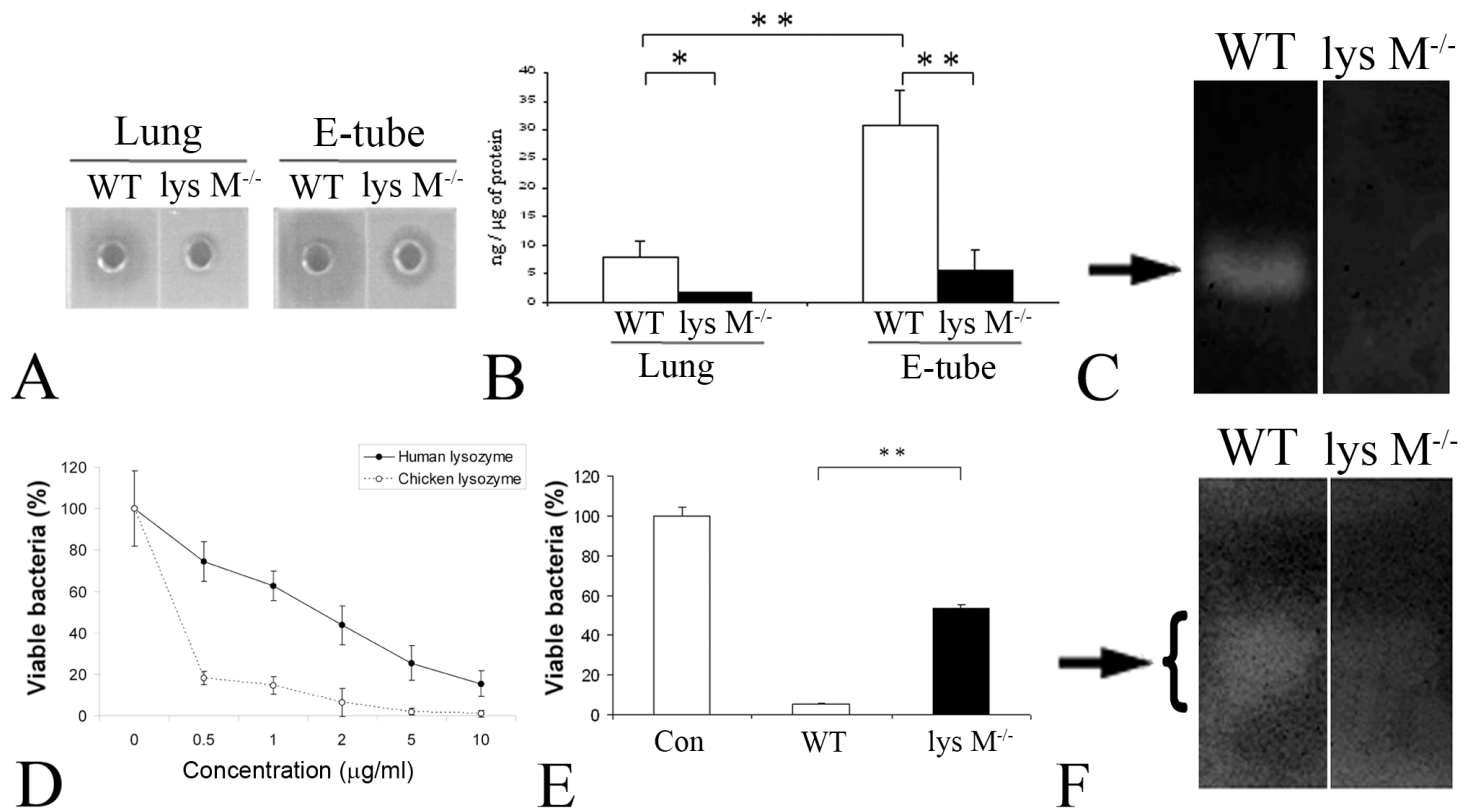
**Depletion of muramidase activity and antimicrobial activity to *S. pneumoniae *6B in the E-tube homogenate of lysozyme M^-/- ^mice**. The lysoplate radial assay of the E-tube homogenate shows depletion of muramidase activity in lysozyme M^-/- ^mice (A). The lysoplate was containing lyophilized *Micrococcus lysodeiktikus*, a substrate of muramidase. The muramidase activity of the tissue homogenate (1 μg) is expressed relative to that of a chicken egg white lysozyme as a standard (B). The gel-overlay assays with the lysoplate demonstrate that the E-tube homogemate of lysozyme M^-/- ^mice lacks muramidase activity (arrow) at the corresponding level of lysozyme (C). The liquid broth assay shows antimicrobial activity of human and chicken lysozyme against *S. pneumoniae *in a dose-dependent manner (D). Antimicrobial activity of the E-tube homogenate decreases in lysozyme M^-/- ^mice, compared to wild type mice (E). 2.5 × 10^4 ^CFU/mL of *S. pneumoniae *6B were incubated with or without the E-tube homogenate (100 μg/mL) for 2 hours at 37°C. Con: the vehicle control, incubated without the E-tube homogenate. Values are given as mean ± standard deviation. All experiments were triplicated. *: p < 0.05, **: p < 0.01. The gel-overlay assays with the bacterial lawn of *S. pneumoniae *6B demonstrate that antimicrobial activity of the E-tube homogenate (arrow) is decreased in the lysozyme M^-/- ^mice compared to the wild type mice (F).

To examine the muramidase activity of the E-tube homogenate, the lysoplate radial diffusion assay was performed, which contained lyophilized *Micrococcus lysodeiktikus*, a substrate of muramidase. The E-tube homogenate of lysozyme M^-/- ^mice showed depletion of muramidase activity (Figure 3A). The muramidase activity of the tissue homogenate (1 μg) was converted to that of a chicken egg white lysozyme as a standard (Figure 3B). Interestingly, the E-tube homogenate contained higher amount of lysozyme per same total protein, compared to the lung homogenate. Moreover, the gel-overlay assay with the lysoplate gel showed that the E-Tube homogenate of the lysozyme M-/- mice lacks muramidase activity at the corresponding level of lysozyme (Figure 3C).

To examine the antimicrobial activity of tissue homogenates, the liquid broth assay was performed. 2.5 × 10^4 ^CFU/mL of *S. pneumoniae *6B was incubated with the purified lysozyme and the E-tube homogenate (100 μg/mL) for 2 hours at 37°C. Human and chicken lysozyme showed antimicrobial activity of against *S. pneumoniae *in a dose-dependent manner (Figure 3D). *S. pneumoniae *6B was inactivated by 94% when incubated with the wild type E-tube homogenate (Figure 3E). In contrast, only 46% of *S. pneumoniae *6B was inactivated, when incubated with the lysozyme M^-/- ^E-tube homogenate (*p *< 0.01). The gel-overlay assay with the bacterial lawn of *S. pneumoniae *6B demonstrated antimicrobial activity of the E-tube homogenate decreased in the lysozyme M^-/- ^mice at the corresponding level of lysozyme (Figure 3F).

### Susceptibility to *S. pneumoniae*-induced OM of lysozyme M^-/- ^mice

**Figure 4:**
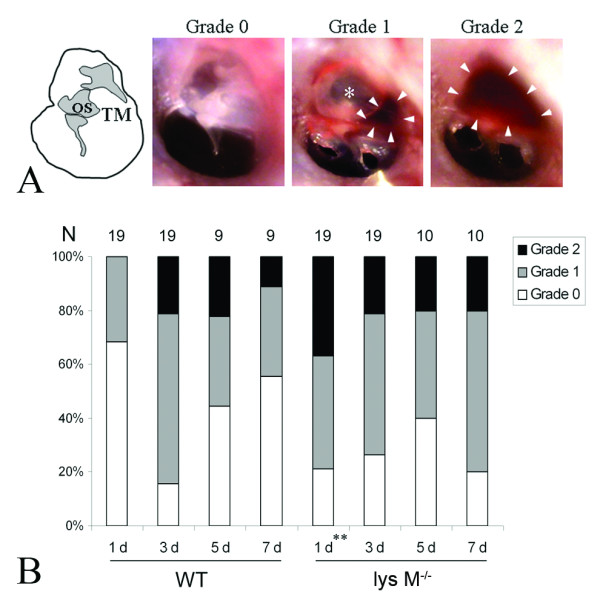
**Depletion of lysozyme M affects the course of middle ear inflammation**. The illustration of the mouse tympanic membrane and the otoscopic views show clinical grades (0, 1 and 2) of the middle ear inflammation (A). OS: ossicles. TM: tympanic membrane. *: the epitympanic space with air-fluid level. White arrow heads: middle ear effusions. The otoscopic findings show that the middle ear inflammation is severe at 1 day after inoculation of live *S. pneumoniae *6B in lysozyme M^-/- ^mice, compared to wild type mice (B). However, the later clinical courses are not different between wild type and lysozyme M^-/- ^mice. This indicates that lysozyme M plays an important role at the early phase of infection, corresponding with the bacterial clearance result (see Table 2). N: number of animals. **: Fisher Exact Test, p < 0.01.

To determine if lysozyme M deficiency increases susceptibility to pneumococcal middle ear infection, live *S. pneumoniae *6B was transtympanically inoculated to the mouse middle ear. After otoscopic observation and photography of the tympanic membrane, the middle ear lavage was plated on chocolate agar plates and colonies were counted after overnight incubation at 37°C in 5% CO_2 _in order to quantify live bacteria. Within one day after inoculation, most of the mice began to show inflammation, characterized by a small amount of turbid and yellow fluid in the middle ear cavity. Compared to wild type mice, bacterial clearance from the middle ear of lysozyme M^-/- ^mice was significantly delayed on day 3, but not on day 7 (Table 2). On day 3, eight out of 9 lysozyme M^-/- ^mice showed positive bacterial culture on day 3, but none of 10 wild type mice. In contrast, only one out of 10 lysozyme M^-/- ^mice showed positive bacterial culture on day 7, but none of 9 wild type mice. The otoscopic findings (Figure 4A) showed that the middle ear inflammation is severe one day after inoculation of live *S. pneumoniae *6B in lysozyme M^-/- ^mice, compared to the wild type mice (Figure 4B). On day 1, Grade 0 (no inflammation) was seen in 13 (68.4%) out of 19 wild type mice, but only 4 (21.1%) out of 19 lysozyme M^-/- ^mice. Grade 2 (severe inflammation with abundant effusion) was not seen in wild type mice, but 7 (36.8%) out of 19 in lysozyme M^-/- ^mice. However, the later clinical courses (on day 3, 5 and 7) were not different between wild type and lysozyme M^-/- ^mice. Taken together with bacterial clearance data, this indicates that lysozyme M plays an important protective role at the early phase of infection.

**Table 2:** Bacterial clearance from the middle ear.

Post-inoculation day	Positive Bacterial Culture*				
	
	Wild type		Lysozyme M^-/-^		Fisher's Exact Test
3	0/10	1+: 0	8/9	1+: 2	*p *< 0.01
		2+: 0		2+: 3	
		3+: 0		3+: 2	
		4+: 0		4+: 1	

7	0/9	1+: 0	1/10	1+: 1	NS
		2+: 0		2+: 0	
		3+: 0		3+: 0	
		4+: 0		4+: 0	

*: The middle ear lavage was collected at 3 and 7 days after inoculation of live bacteria to investigate the presence of live bacteria. Values are given as the number of culture positive animals/total number of animals. NS: no significant difference. 1+: 1 ~ 10 colonies, 2+: 11 ~ 20 colonies, 3+: 21 ~ 30 colonies, 4+: > 30 colonies.

## Discussion

In this study, we demonstrated that lysozyme M deficiency results in a decrease in both muramidase activity and antimicrobial property of E-tube in spite of compensatory up-regulation of lysozyme P.

Moreover, we showed for the first time that lysozyme M^-/- ^mice were more susceptible to pneumococcal OM with severe inflammation compared to wild type mice, especially in the early phase. It was hardly expected that lysozyme M deficiency resulted in delayed clearance of *S. pneumoniae *from the middle, considering lysozyme M is just one of the anitimicrobial molecules.

Our result suggests that lysozyme M plays a critical role as a major antimicrobial molecule of the E-tube against *S. pneumoniae *before induction of host adaptive immune system. Experiments are currently underway with over-expressing lysozyme M mice to see if these transgenic mice are more resistant to infection.

Although introduction of antibiotics has led to a decrease in the incidence of life-threatening complications of OM [26], the rapid emergence of antibiotic resistant OM pathogens requires an alternative strategy for management of OM [27]. AIIMs are considered as a possible alternative therapeutic approach, which could overcome current trends of increasing antibiotic resistance among OM pathogens. There are evidences demonstrating bacterial countermeasures to AIIMs, such as covalent modification of bacterial wall and/or membrane and expression of membrane proteases destroying AIIMs [28-31]. However, it is likely that bacteria have not generally developed effective resistance to AIIMs, considering AIIMs have been around for a long time in the animal kingdom.

Lysozyme is highly expressed in the tubotympanum of all the species examined, such as guinea pigs, chinchillas, rats, and mice [9] and increases with age in both humans [32] and in mice [33]. It is also present in the middle ear effusion of OM patients although at higher concentrations in the mucoid effusion compared to the serous effusion [34,35]. The presence of lysozyme in serous cells of the tubal glands and the secretory epithelial cells suggests a role for this molecule in the defense of the middle ear and E-tube against pathogens[9,32,34].

Lysozyme is a 14 kD cationic enzyme, hydrolyzing the β (1–4) glycosidic bond between the two major repeating components of peptidoglycan: N-acetyl muramic acid and N-acetyl glucosamine [36]. Although there is a debate about the mechanism of lysozyme's antimicrobial activity such as muramidase-dependent and -independent mechanisms [37,38], it is believed that the muramidase activity of lysozyme plays a critical role in inducing inflammation of hosts by production of peptidoglycan fragments [39]. For the muramidase-independent antimicrobial mechanism, it is known that the cationic helix-loop-helix structure of lysozyme is important [40].

In mammals, lysozyme can be found in most tissues and is an important component of the innate defense system of all mucosal surfaces, including the digestive tract, genitourinary tract, the respiratory tract, as well as the E-tube and the middle ear [9-12]. Lysozyme over-expression leads to an increased survival rate after streptococcal and pseudomonal lung infection [41], while lysozyme deficiency results in a decrease in pseudomonal clearance from the airways [17] and an increase in micrococcal skin inflammation [16]. Consistent with these findings, our result also showed that lysozyme deficiency results in an increased susceptibility to pneumococcal OM in mice.

In mice, there are two types of lysozyme: lysozyme M and lysozyme P as mentioned earlier. The 130-amino-acid mature peptides of both lysozyme M and lysozyme P differ by only 11 amino acid substitutions [16], which results in differences in electrophoretic migration and antimicrobial activity. Lysozyme M and lysozyme P show similar bactericidal activity against Gram-positive bacteria, whereas lysozyme M is more effective than lysozyme P in killing Gram-negative bacteria [42]. We here focused on lysozyme M because it is a predicted orthologue of human lysozyme. We previously demonstrated that human lysozyme reduces the viability of a major OM pathogen such as *S. pneumoniae *6B in a dose-dependent manner and acts synergistically with β defensin-2 [43], another very important member of AIIMs. Lysozyme alone is not very effective against Gram-negative bacteria, while lysozyme, in combination with lactoferrin and defensin is bactericidal against several Gram-negative strains such as *Haemophilus influenzae *[9,44].

Lysozyme P is normally inactive in macrophages, but lysozyme M gene deletion results in a compensatory increase of lysozyme P expression [16,42]. The disruption of the lysozyme M gene may alter the DNA conformation at the locus, resulting in aberrant transcription of genes from regions that are downstream of the lysozyme M locus, some of which are regulatory [45]. However, further studies are necessary to elucidate the underlying molecular mechanisms. Our results showed that lysozyme P is up-regulated in the E-tube of lysozyme M^-/- ^mice, compensating lysozyme M depletion, but it apparently seems to be insufficient for protection. We demonstrated up-regulation of lysozyme P mRNA in the lysozyme M^-/- ^mice, but lysozyme P protein activity (muramidase or antimicrobial activity) was not apparent in the protein concentration range (< 0.2 mg/ml), used in this study. Moreover, lysozyme was labeled only in the submucosal glands of the lysozyme M^-/- ^mice, indicating predominant expression of lysozyme P in the submucosal glands, not in the tubal epithelium. It is suggested that lysozyme M and lysozyme P are differentially expressed, specific to some non-ciliated epithelial cell types of the E-tube, but further study is necessary. Finally our result showed that lysozyme M-independent muramidase or antimicrobial activity was insufficient for compensate lysozyme M deficiency, suggesting lysozyme M is most abundant in the basal, non-stimulated state compared to other AIIMs, which are usually inducible upon exposure to pro-inflammatory stimulation.

## Conclusion

Taken together, it is evident that lysozyme M plays a critical role in protecting the middle ear against invading pathogens, especially in the early phase. We suggest that exogenous lysozyme could be used as an adjuvant therapeutic agent for treating OM; however, further studies are necessary to test this hypothesis.

## Abbreviations

OM: otitis media; E-tube: Eustachian tube; AIIM: antimicrobial innate immune molecule; AU-PAGE: Acid-Urea polyacrylamide gel electrophoresis.

## Competing interests

The authors declare that they have no competing interests.

## Authors' contributions

JS performed most of experiments. SKM designed experiments, analyzed data and prepared the manuscript. HYL prepared the lysate of NTHi, and assisted experiments related with bacteriology. TT assisted experiments related to radial diffusion assay and gel overlay assay. HP assisted experiments related immunoblotting and immunohistochemistry. JIW assisted experiments related real time quantitative PCR. RG assisted analyzing data and assisted preparing the manuscript.

NY oversaw study design and analysis. DJL is recipient of DC005025 and DC006276, which supported this work and the studies were conducted in his laboratory, with him as the principal investigator.

## Pre-publication history

The pre-publication history for this paper can be accessed here:


